# COVID-19 vaccine refusal as unfair free-riding

**DOI:** 10.1007/s11019-023-10188-2

**Published:** 2024-01-08

**Authors:** Joshua Kelsall

**Affiliations:** University of Warwick, PAIS Building, Coventry, CV47AL UK

**Keywords:** COVID-19, Vaccine hesitancy, Free-riding, Fairness, Vaccines

## Abstract

Contributions to COVID-19 vaccination programmes promise valuable collective goods. They can support public and individual health by creating herd immunity and taking the pressure off overwhelmed public health services; support freedom of movement by enabling governments to remove restrictive lockdown policies; and improve economic and social well-being by allowing businesses, schools, and other essential public services to re-open. The vaccinated can contribute to the production of these goods. The unvaccinated, who benefit from, but who do not contribute to these goods can be morally criticised as free-riders. In this paper defends the claim that in the case of COVID-19, the unvaccinated are unfair free-riders. I defend the claim against two objections. First, that they are not unfair free-riders because they lack the subjective attitudes and intentions of free-riders; second, that although the unvaccinated may be free-riders, their free-riding is not unfair.

## Introduction

COVID-19 vaccination programmes promise several valuable goods. First, they can create herd immunity, which promises to slow the spread of the virus; reduce the risk of new variants; and protect those who cannot get immunity through vaccination. Second, they reduce the strain on health care systems struggling to provide normal services and cope with a mass influx of COVID-19 patients. Third, they can allow governments to lift restrictions on freedom of movement, such as quarantine or home working with a reduced risk of outbreaks.

By getting vaccinated, people can contribute towards these goods. However, people may benefit from these goods without being vaccinated. A moral criticism of the unvaccinated is that they are free-riders: they unfairly benefit from vaccination programmes without contributing.

Academics have debated whether vaccine hesitancy is, in general, free-riding (Bauch et al. 2010; Bradley and Navin 2021, 2022; Betsch et al. 2013, 2017; Buttenheim and Asch 2013; van den Hoven 2012; May and Silverman 2005; Schröder-Bäck et al. 2009; Verweij 2022; White 2021).^1^ However, whether it is free-riding depends in part on the value of the goods created through vaccination, which will vary depending on the vaccine and the disease in question. As such value varies depending on the case, I focus on COVID-19 as a paradigm case where vaccine refusal is unfair free-riding. I argue that COVID-19 vaccine refusal is morally unfair free-riding.

In making my argument, I also respond to two counter-arguments. The first, from Bradley and Navin, is that vaccine refusal cannot be free-riding of any kind, because vaccine refusers often fail to have the subjective attitudes and intentions of free-riders. I argue that the subjective intentions and attitudes of vaccine refusers are irrelevant to whether they free-ride. The second counter-argument, from Verweij, accepts with me that vaccine refusal is free-riding, but rejects the claim that such free-riding is unfair. I give three counter-arguments which show that COVID-19 vaccine refusal is unfair free-riding.

Section "Unfair free-riding", introduces the characterisation of unfair free-riding. In Section "COVID-19 vaccine refusal as unfair free-riding", I characterise COVID-19 vaccine refusal as unfair free-riding. In Section "Objection: subjective free-riders", I respond to Bradley and Navin’s (2021, 2022) arguments that vaccine refusal is not free-riding. In Section "Objection: vaccine refusal is free-riding, but not unfair", I respond to Verweij’s (2022) argument that although vaccine refusal is free-riding, it is not unfair.

## Unfair free-riding

I define free-riding from a moral perspective.^2^ On this perspective, free-riding is immoral due to its unfairness (Cullity 1995, p. 1). The kind of unfairness is a failure of appropriate impartiality, where the free-rider makes an unjustified exception of herself by failing to pay her share towards a collective good (Trifan 2020, p. 159). A good is collective if it satisfies the condition of *jointness in* supply; this is the condition that “if a public good is available to one member of the group for which it is public, then it is available to every other member at no cost to that other member” (Cullity 1995, p. 3; Trifan 2020, p. 167).

A theory of moral free-riding will provide the conditions under which it is wrong for a putative free-rider to excuse themselves from paying their fair share towards a collective good. Here is a general formulation of unfair free-riding: If some individual or group X produces a collective good φ, and some other individual or group Y benefits from φ without paying a reasonable cost for φ, then Y is a free-rider.^3^

This principle is too strong without modification. Nozick claims that “one cannot, whatever one’s purposes, just act so as to give people benefits and then demand payment. Nor can a group of persons do this” (Nozick 1974, p. 95). Nozick is concerned that the principle gives the right to obligate others to pay for benefits regardless of whether the benefit was desired or solicited by the beneficiary (Cullity 1995, p. 5; Nozick 1974, p. 95). While this concern is correct, his own position is too strong (Arneson 1982, pp. 617–618). It is true that nobody has the unrestricted right to coerce others by bestowing collective goods on them and then demanding reciprocal payment; however, there are cases where it would be unfair for Y to benefit from a collective good φ produced by X without paying reasonable costs for φ. Thus, we need a more restrictive principle of fairness. Consider these cases.

*Fare Evasion*

Public transport in my town is efficiently run on an ‘honour’ system, which places the onus on passengers to buy a ticket before travelling and to cancel it in a machine on any vehicle they use. I ride without paying (Cullity 1995, p. 5).

*The Enterprising Elves*

Cryfield Road is a private road that is strewn with litter and scrap from its slovenly residents. One morning, the residents are delighted to find their immaculate, having been cleaned by some enterprising elves. They are less than thrilled when the elves come knocking at their doors that morning with the bill.^4^

In *Fare Evasion,* I unfairly benefit from the good produced by others who pay their fares while I do not. Conversely, in *Enterprising Elves*, it is unreasonable for the elves to demand payment for their work, even though the residents benefit in having their road cleaned. The elves may deserve gratitude for doing something so nice; but niceness doesn’t give anyone the right to demand a material reward for their niceness. Prima facie, the neighbour’s lack of voluntary consent to the elves’ work is what makes it wrong for them to demand payment, (however reasonable the payment may be) for their work. However, voluntary consent can’t fix the principle of fairness; there are cases where Y may not voluntarily consent to benefit from a good, but their behaviour remains unfair. Consider an alternative private road case:

*Private Road*

John lives on Cryfield road, which is now rundown with potholes. It is damaging everyone’s cars, and the community wants the road fixed. John is a cheapskate, even though he knows he can well afford it and would pay for it if came to it. However, he also knows that if he refuses to pay, his neighbours will pay to fix the road anyway, so he refuses. John also has no choice but to use the road since his house is near the end of the road.

John does not voluntarily consent to contribute to or benefit from the scheme; regardless, he will benefit as he lives on the road. Yet his behaviour is paradigmatically unfair free riding. It is unfair of him to benefit from the fixed road at his neighbours’ expense when he could have paid his share. Thus, our fairness principle must explain why it can be unfair not to contribute to a scheme even when voluntarily consent is lacking.

Such a principle is required for COVID-19 vaccine refusal. Herd immunity is an example of a good in which we have what Dawson calls a convergent interest. Goods in which we have convergent interests cannot be broken up into individual goods without destroying the good itself (2011, p. 15).^5^ As herd immunity only exists as a good when enough people co-operate by getting vaccinated, it is not a good that an individual can possess solely by making and individual contribution. Moreover, because not everyone needs to co-operate to produce the good (only a threshold level of immunity is needed), Dawson notes that goods that are in our convergent interests are ripe for free-riding, since it is possible to benefit for at least some to benefit from the good without contributing to its creation or maintenance (ibid). Lastly, one can stand to benefit from the good of herd immunity irrespective of whether one consents to contributing to its production. Therefore, if voluntary consent is necessary for unfair free-riding, vaccine refusers aren’t free-riders. Insofar as we take their behaviour to be unfair, we need a principle of fairness that explains why free-riding is unfair even in (at least some) cases where the free-rider doesn’t consent to the co-operative scheme or the good it produces.

Cullity proposes the following principle of fairness that characterises unfair free-riding without voluntary consent (1995, pp. 18–19). If some individual or group X produces a good φ; and if some other individual or group Y benefits from φ without paying a reasonable cost for φ; and if the following three conditions hold, then Y is an unfair free-rider.

1. Paying for φ represents a net benefit to Y that is worth its cost.
2. A fair generalisation of paying for goods in similar cases to φ would not leave practically everyone worse off.
3. There are no legitimate moral objections against φ or paying for φ.

Regarding (1), Cullity states that a good provides a net benefit if it is ‘all things considered’ worth its cost and its costs are shared fairly (1995, pp. 16–18). In determining whether a good is ‘all things considered’ worth it’s cost, we can use the value of the good in question. Klosko distinguishes between presumptively beneficial goods and discretionary goods. The former would ground obligations to contribute to their production because they are beneficial to practically everyone who has them; while the value of discretionary goods is variable depending on preferences (Klosko 1987; Trifan 2020, p. 161). Klosko’s thought is that when a good is presumptively beneficial, one is obligated to contribute to the production of such a good because it is fundamentally in one’s interest to benefit from such goods, whereas when it comes to discretionary goods, this is not necessarily the case because the value of such goods is contingent on personal preference.

Costs are shared fairly if they are shared equitably, and if they do not make contributors worse off than they would have been if they didn’t contribute. Clearly, there is a vagueness about when benefits are worth their costs. Suppose in *Private Road*, John does not drive, and mostly uses an alleyway instead of the road, then perhaps repairing the road is not worth the cost for him. Of course, John still benefits from the fixed road, and his neighbours could propose a more reasonable cost given his infrequent usage. Thus, we can change whether a benefit is worth its cost by negotiating its cost. To know whether a benefit is ‘all things considered’ worth its cost, we need to look at the benefit itself. Here, we can distinguish between luxury benefits and more fundamental benefits. Suppose John’s neighbours decide to keep the road clean, which requires each neighbour to spend ten minutes a week sweeping it. While this would benefit John, and the cost is minimal, the benefit is a luxury that is ultimately insignificant to John. However, in the original case, the road is damaging John’s car and lowering the value of his home; therefore, there are good reasons to think that the benefit is more necessary to John’s wellbeing, and so worth paying a reasonable price for.

Regarding (2), we must consider whether generalising the demands of the case would leave the community worse off as a whole (Cullity 1995, p. 14). Take *Enterprising Elves*. A fair generalisation of this case would create an economic system where agents are obliged to pay for any unsolicited good that provides a collective benefit. This generalisation would “be so cripplingly inefficient that it would impoverish us: it is clearly better for practically everyone if commercial transactions can only be entered into by means of an explicit act of commitment” (Cullity 1995, p. 14).^6^ Condition (2) addresses Nozick’s worry that principles of fairness give people a general right to demand payment simply by providing benefits. It does so by restricting us to those cases where a general right to coerce others to pay for goods from which they benefit would not leave people overall worse off.

Condition (3) requires that the good to be contributed to, and the means by which it is contributed to, be morally unproblematic. Moral problems could occur if the good or the means of contributing to it involve injustice, or if they are wasteful, cruel, or degrading (Cullity 1995, p. 19). Suppose John’s neighbours decide to pay a group of first-generation immigrants to fix the road because they can underpay them; or suppose they decide to pressure Margaret, a non-driving, but wealthy old lady on the street, to pay for an unfair portion. Although John could benefit from both schemes, it is not unfair to refuse to contribute because these schemes are morally objectionable.

*Fare Evasion* and *Private Road* satisfy all three conditions. In *Fare Evasion,* everyone benefits from the honour system, since it keeps a valuable public service running; the cost is distributed fairly (assuming everyone is required to pay an equal and reasonable cost for the train ticket); a fair generalisation requiring people to pay their fares for the public transport systems they use wouldn’t leave them worse off; and there are no obvious moral objections to the system. In *Private Road*, the neighbours all benefit from not having their cars ruined by the damaged road and the cost is distributed fairly (they take into account differences in income and road usage); a fair generalisation requiring people on private roads to pay for their upkeep would not leave everyone on private roads worse off; and there are no obvious moral objections against fixing one’s damaged road when one can afford it.

*Enterprising Elves* fails all three conditions. The elves provide a collective benefit and may suggest a reasonable cost; however, the benefit is a luxury benefit and so the mere fact that the resident are pleased with the nice view from their windows is insufficient to require them to pay the elves, though they may well be grateful. We already showed that fairly generalising the enterprising elves’ business model would leave practically everyone worse off. Lastly, there is a legitimate moral objection as the elves have tampered with the resident’s property without their permission.^7^ Thus, consent may play a role as a legitimate moral objection to specific cases, even if it isn’t a necessary condition for unfair free-riding.

On Cullity’s definition of unfair free-riding, people can be liable to contribute to goods without their voluntary consent. If the good provides a net benefit; if a fair generalisation of contributing to the benefit would not leave practically everyone worse off; and if one is not raising legitimate moral objections to the public good, then one is liable to pay a reasonable cost for that good. If one refuses to do so while benefitting from that good, then one is an unfair free-rider.

## COVID-19 vaccine refusal as unfair free-riding

In this section, I apply Cullity’s characterisation of unfair free-riding from Section "Unfair free-riding" to COVID-19 vaccine refusal. I argue that such refusal satisfies the three conditions of unfair free-riding.

First, we must identify the goods gained from contributing to a COVID-19 vaccination programme. The most obvious is herd immunity. Herd immunity occurs when the immunity of a large proportion of a population creates resistance to the spread of an infectious disease. People become immune either through vaccination or from catching the disease. The level of immunity required is typically between 80 and 90% (WHO 2020). As of 2020, in the UK, vaccination rates for a second dose are 88.3%, and 70.2% for a third dose (UK Health Security Agency 2023). Whether this reaches the threshold for herd immunity, given the lower proportion of people with the third dose, is unclear. We don’t know for certain the overall level of immunity; that would require us to determine what proportion of the population gained immunity by contracting the virus in the wild. However, the UK was at least close to herd immunity threshold in 2020, and there are still goods gained from a high vaccination rate, as I discuss below.

Herd immunity is a *public* good. Goods are public to the degree that they satisfy the criteria for publicity. Relevant for our purposes are two of these: non-excludability and nonrivalness (Cullity 1995, pp. 6–7).^8^

*“Nonexcludability*: if anyone is enjoying it, no one else (in the group for which it is public)^9^ can be prevented from doing so without excessive cost to the would-be excluders.

*Nonrivalness*: one person’s enjoyment of the good does not diminish the benefits available to anyone else from its enjoyment.”

Herd immunity is nonexcludable and nonrival (Buttenheim and Asch 2013; Ivankovic and Savic 2022; Fisman and Laupland 2009; White 2021; van den Hoven 2012). If a sufficient proportion of the population is immune from COVID-19, then it is impossible to exclude anyone from benefiting from herd immunity; at least not without the substantial cost of forcing the unvaccinated to become infected.^10^ The good is also nonrival because one person’s enjoyment of herd immunity does not prevent another’s enjoyment of it to the same degree. Because herd immunity is nonexcludable, unvaccinated people benefit from herd immunity regardless of their consent. However, this need not worry us because voluntary consent is not required to obligate one to contribute towards the production of goods.

Discussions of free-riding in the context of vaccine refusal often focus on herd immunity as the primary public good gained through community vaccination (Bradley and Navin 2021, 2022; van den Hoven 2012). However, community vaccination creates other public goods, at least in the case of COVID-19. We should mention these goods since they will affect the overall net benefit gained through a successful vaccination programme. White (2021) presents three additional public goods. First, the reduction of strain on public health systems that otherwise would be tested by Covid. Consequences of COVID-19 on the NHS in the UK include inability to provide adequate healthcare; increased waiting lists for vulnerable patients; delayed diagnoses; and overwork and stress on NHS staff.^11^ Second is the public good for the removal of social and workplace restrictions. Third is the slowing down of new variants of the virus.^12^

The benefits of public freedom created by removing restrictions, is nonexcludable, as are the benefits of the NHS, at least for those with the right to reside in the UK who have the right to use the service and have obligations to support it through taxation.^13^ Although it is theoretically possible to exclude the unvaccinated from public spaces or the NHS, doing would be costly.^14^ While implementing a passport system is possible, this would impose heavy implementation and maintenance costs. Furthermore, if the passport scheme is to be just, it must accommodate unvaccinated individuals who are immune from COVID-19 through contracting the virus (Shenai et al. 2021). The scheme must be just or the unvaccinated would have a legitimate moral objection to it, and so would not be free-riding if they refused vaccination. However, implementing a system to incorporate those who are immune through infection adds complexity and thus cost to the strategy. Health promoted through the NHS is largely nonrivalrous, though where its capacity is stretched, as it was during the pandemic, it can become rivalrous. The general freedom of movement gained by removing restrictions is nonrival as everyone can enjoy this benefit without diminishing anybody else’s enjoyment of it.

The benefits of herd immunity and a lower rate of variants are nonexcludable and nonrival to a higher degree than the previous public goods. Still, there are exceptions. Unvaccinated people sometimes live in geographic clusters. This means that they are more prone than people living elsewhere to suffer from the virus and new variants (Alvarez-Zuzek et al. 2022). Though they may still benefit from herd immunity and lower variant rates, they benefit less, and so they may not count as free-riders on these goods. Still, there may be other goods in such communities that provide net benefits, such as the removal of restrictions. And as White notes, there are benefits “by slowing the spread (and resultant consequences of infection) to some degree… herd immunity is not an all-or-nothing proposition” (White 2021, p. 2; Yates 2021).

I have shown what goods can be gained through a successful vaccination programme. Now we can ask whether vaccine refusers satisfy Cullity’s conditions for unfair free-riding.

First, the public goods discussed above provide net benefits and their costs are fairly distributed. As Sorell notes, pandemics such as COVID-19 “impair the long-term local provision of a social minimum—even by rich governments that eventually gain the co-operation of their publics” (forthcoming: 10). The idea of a social minimum refers to a basic bundle of resources that is required for persons to live a minimally decent life in society (White 2021).^15^ Further to the damages to public health services, is damage to services such as transportation, education, and social care. Indeed, the prolonged pandemic led to mass school closures. This resulted in defects in childhood and adolescent education, social, linguistic, mental, and even physical development (Chaabane et al. 2021; Viner et al. 2021); poorer social care for vulnerable people, be it elderly people in nursing homes resulting in negative mental health outcomes (Jones et al. 2022) or women suffering domestic violence unable to access shelters (Piquero et al. 2021); disruption of transport services (Subbarao & Kadali 2022); and significant damage to the economy affecting people’s ability to both earn a living and afford to pay for their basic needs (Shanmuga et al. 2021). In the UK, at the start of the pandemic, the number of people in work fell by 825,000 throughout 2020, and the number of economically inactive people rose by 327,000; job redundancies reached record highs, and working hours dropped to their lowest since 1994 (Francis-Devine et al. 2022). The most affected groups were ethnic minority groups, men, the youngest and oldest workers, and low paid workers; in some cases, this is because they were more likely to be made redundant or because they were unable to access their workplaces due to restrictions (Francis-Devine et al. 2022).

Sen’s description of the basic social goods constituting a social minimum is that it involves the “beings and doings [which]…can vary from such elementary things as being adequately nourished, being in good health, avoiding escapable morbidity and premature mortality, etc., to more complex achievements such as being happy, having self-respect, taking part in the life of the community, and so on” (Sen 1992, p. 39). Damages to the proper function of healthcare systems during COVID-19, (and COVID-19 itself) is an imminent threat both to good health and avoiding morbidity and premature mortality. Maintaining a stable income that allows one to afford basic goods is necessary for happiness and self-respect, yet these were undermined by the pandemic, creating an unstable job market, closing businesses, and redundancies, often on people who were already economically vulnerable pre-pandemic. The possibility to take part in a community is dependent on education and the ability to participate in social activities in a community; the pandemic threatened these by resulting in the closing of schools, transport services, and limiting social contact, resulting in educational deficits in children and adolescents, damaging the mental health of elderly people, and restricting the opportunities vulnerable women (and other victims of violence) access to services that might support them. In light of this discussion, we can reasonably conclude that in threatening these basic social goods, COVID-19 was a threat to the social minimum.

Second, immunisation schemes typically distribute their costs fairly. For instance, the UK government offered everyone access to the vaccines. When it gave special priority to some groups, this was justified. For example, those most vulnerable to covid, such as the elderly and people with pre-existing health conditions, were offered the vaccine before younger, healthier people. Also, exceptions were made for those whom the vaccines were not known to be safe until relevant studies had been done; for example, pregnant women (Satin and Sheffield 2022).

Third, these benefits are worth the costs. Not only for the reasons already given, but because the costs of vaccination to individuals are minimal; severe reactions to covid vaccines occur in 1–2 people for every million doses given (US Department of Health & Human Services 2022). Moreover, the inconveniences of accessing the vaccine are insignificant compared to the costs of being unvaccinated and the benefits of a successful vaccination programme. Thus, the costs of vaccination are overwhelmingly worth their benefits.

To meet Cullity’s second condition, we must show that practically no-one would be worse off if we fairly generalised a norm to contribute to vaccination programmes for other infectious diseases, such as the annual flu, measles, mumps, and rubella, for example. Prima facie, such a generalisation would leave practically everyone worse off. A norm making everyone liable to vaccinate against many or all infectious diseases would impose unfair demands, even if everyone would be better off by being less susceptible to these diseases. We can avoid this worry by distinguishing COVID-19 from most other infectious diseases. In doing so, our fair generalisation will only include infectious diseases with similar features to COVID-19. Such a fair generalisation of these cases would not leave practically everyone worse off.

My position rests on independent, but related, arguments made by Sorell and Vanderheiden. Vanderheiden argues that people are liable to contribute to schemes mitigating the effects of climate change. Consent to the scheme is not necessary for liability to contribute because the public good of a stable environment is fundamental to the non-basic goods that we enjoy.

“Since the activities responsible for excessive carbon emissions are non-basic… and since the non-basic good… that allows persons to compromise this basic good is likewise of a lower priority in this hierarchy, requiring fair contributions toward a total provision of the more basic good may be justified by the priority of basic over non-basic goods” (Vanderheiden 2016, p. 20).

For Vanderheiden, the free-rider privileges non-basic goods above their own and others’ basic public goods (2016, p. 21). It is in this sense that free-riding counts as unfair. We may understand Vanderheiden’s distinction between basic goods and non-basic goods as similar or analogous to Klosko’s distinction between presumptively beneficial and discretionary goods.

Sorell’s argument doesn’t concern free-riding; he is concerned with the justification for regulating free speech to prevent the spread of COVID-19 conspiracy theories. He argues that the threat to a social minimum caused by conspiracy theories justifies restrictions on free speech. We needn’t go into the details of his argument for this claim; what is of note is that Sorell faces a similar problem to our own. If suppressing COVID-19 conspiracy theories is justifiable to prevent deaths caused by COVID-19, why isn’t it justifiable to suppress all conspiracy theories about infectious diseases? There are two aspects to Sorell’s response. First, the threat of COVID-19 to a social minimum: that is, a threat to a basic bundle of resources required for persons to live a minimally decent life in society (forthcoming: 10). Not every infectious disease presents such a threat, because either the disease is not overly serious; it is under control; or the NHS is well equipped to deal with it. It is this point about the social minimum that connects with Vanderheiden’s point about basic social goods. However, Sorell’s argument is stronger since the absence of some basic goods may not undermine a social minimum. For example, endemic infectious diseases such as influenza do not present an overwhelming threat to health care systems: routine vaccinations of vulnerable patients, strong immune systems in the general population, means that the disease does not threaten the provision of healthcare or any other basic goods. Even diseases that break out in geographic clusters, such as measles breaking out in a geographic cluster of anti-vaxxers, does not constitute a pandemic threatening the provision of a basic social minimum in society. Moreover, in such cases, the harm caused is a direct result of the anti-vax attitudes of those who do not vaccinate; if almost everyone outside the cluster is vaccinated, then there is no one to unfairly free-ride, and no one to morally criticise for the outbreak aside from the unvaccinated. Thus, in these cases, we would not require a norm of vaccination on grounds of unfair free-riding since these diseases do not pose the substantial threat to a social minimum that the COVID-19 pandemic did.

Sorell (*forthcoming*) notes a further distinction between COVID-19 and other infectious diseases. Not only does COVID-19 present a threat to a social minimum, but at the start of the pandemic at least, this type of threat was an emergency. In earlier work, he defines an emergency as a situation that requires an urgent response from appropriately placed agents if severe harm is to be completely avoided or reduced (Sorell 2013, chp.2) In public emergencies, many people in a community face such harm and governments or public institutions are the relevant agents (ibid). For Sorell, the emergency status of the COVID-19 outbreak combined with its being a threat to a basic social minimum, justifies restrictions on free speech. In our context, the distinguishing features of COVID-19 are that it threatens a social minimum and that it is an emergency, and this is sufficient to distinguish it from other cases of infectious diseases, such as those that are endemic, confined to geographic clusters, and well within the capacities of health care systems to deal with.

Taking the social minimum argument, then, a fair generalisation of our scheme would not make everyone liable to vaccinate against all infectious diseases. It would mean that one is liable to contribute to a vaccination scheme if: (1) this scheme is an effective means to overcome an emergency; and (2) that emergency is a threat to a basic social minimum. This generalisation restricts us to a subset of infectious diseases: those that cause emergencies that threaten a social minimum. This satisfies Cullity’s second condition. It isn’t the case that practically everyone would be worse off by protecting a social minimum in emergencies. It is on this social minimum that the enjoyment of one’s non-basic goods and life plans depends.

Condition (3) requires us to work out whether there are any legitimate moral objections to mass vaccination programmes. In her paper, which also applies Cullity’s notion of unfair free-riding to vaccine refusal but in general terms, van den Hoven argues that there are legitimate moral objections to vaccination schemes, though the number of objections is small (2012, p. 157). First, she discusses vaccine refusal by parents for their children grounded in (false) beliefs about vaccine safety. On Cullity’s definition, these are not legitimate moral objections. While parents believe that vaccination is not a net benefit for their children, this belief is wrong, because based on false beliefs about vaccines (van den Hoven 2012, p. 157).

A stronger moral objection to free-riding comes from certain religious perspectives. The first objection, noted by van den Hoven, is that as religious communities often live together, they can create geographic clusters where herd immunity is less effective if vaccine hesitancy is common in the community. van den Hoven concludes that “due to the geographical closeness of most orthodox religious families that refrain from immunisation, herd immunity is not guaranteed in villages where they live… This could weaken the claim that they free-ride on herd immunity because their net benefit is actually lower than for others” (2012, p. 158). This argument doesn’t quite work, however. First, van den Hoven is treating the vaccination problem generally, and specifically focusing on non-emergency cases. Second, she focuses on herd immunity as the salient public good. However, benefits such as protecting the health service and removing restrictions are felt even in Bible Belt areas, and thus there are still public goods that those in such clusters can share in and contribute to through vaccination.

There are two further kinds of religious objection that we might come up against. The first is grounded in concerns about the compatibility of vaccination with one’s religious precepts. For example, suppose a Muslim is concerned that vaccines are not halal, a legitimate moral concern in some Muslim communities. Suppose that they raise this concern with their faith leader, who shows that the vaccine is halal. In that case, the moral objection is no longer legitimate once it is demonstrably refuted by the relevant member of the faith community. To address this kind of religious moral objections, we must engage with the precepts of the faith and show their compatibility with vaccination. Where this is impossible, it would be wrong to characterise the person as a free-rider. This doesn’t damage the thrust of my argument since such cases are arguably a small minority; most objections to COVID-19 vaccines are based on misconceptions about vaccine safety and effectiveness (Mascellino et al. 2021; Wouters 2021), not intractable moral debates.

The second kind of religious objection is harder to refute. It rests partly on the previous objection since it depends on whether vaccination really is incompatible with religious precepts. If it is not, then we can use the previous strategy. However, assuming that vaccination is incompatible with a religion, a religious person may argue that vaccination is not in their interests, even if it results in death, because a life of religious integrity is more important to them.

In an emergency that threatens to undermine a basic social minimum, we could argue that the enjoyment of religious freedom and expression itself presupposes a basic social minimum. It presupposes a basic social minimum on the grounds that the enjoyment of religious freedom and expression in any robust sense would require for many, the existence of religious institutions, the ability to take part in sacred rituals and ceremonies, to be part of a broader religious community. Given that COVID-19 is a threat to a basic social good, such as human life, even someone interested in enjoying religious freedom requires a basic social minimum to enjoy that freedom. However, a religious person can respond that death is acceptable, given something like a heavenly reward for maintaining their belief in the face of it. In *that* case, we reach an impasse. However, as noted previously, much of vaccine hesitancy is concerned with safety and efficacy, not with these extreme religious cases.

A final point on moral objections. We should distinguish objections to coercive vaccination policies from objections to getting vaccinated. While there may be legitimate objections to coercive vaccination policies, these are distinct from the question of whether one should get vaccinated. This is because there may be political or moral reasons against government coercion, even if there is a moral liability to get vaccinated (van den Hoven 2012). This rules out moral objections that are not about vaccination, but rather about coercive policies that try to enforce vaccination.

Those who benefit from the goods produced by a vaccination programme without supporting the programme are unfair free-riders. They should have contributed to the production of those goods as they provide a net benefit: protection of a social minimum that is worth its cost; the costs of vaccination are distributed fairly; and a fair generalisation of a principle that requires cooperation with schemes that protect a social minimum or basic goods in emergencies does not leave practically everyone worse off. Finally, while there are moral objections against vaccination, many are either illegitimate or refutable. In the rest of this paper, I consider several objections to the view that covid-19 vaccine hesitancy is unfair free-riding and reject them.

## Objection: subjective free-riders

Bradley and Navin claim that vaccine hesitancy is not free-riding. They argue that taken either subjectively or objectively, vaccine refusers are not free-riders. Their objective argument depends on an interpretation of free-riding as a prisoner’s dilemma problem (Bradley and Navin 2021). Because prisoner’s dilemma free-riding differs from unfair free-riding, we can ignore the objective argument.^16^ However, as the subjective argument is separable from prisoner’s dilemma free-riding, I address it here.

Here is Bradley and Navin’s subjective argument:

“[V]accine refusers do not think they are using a valuable public good (community protection) or that they are refusing to make a responsible contribution to that good since they think the expected costs of vaccination are very high” (2021, p. 168).

Bradley and Navin do not focus on COVID-19; they typically focus on parents’ refusal to vaccinate their children from diseases such as measles, mumps, and rubella (MMR). They justify their argument with three points. First, that the reasons given for vaccine refusal are often about the safety and efficacy of the vaccines, not a desire to benefit from herd immunity (2021, p. 171; Harmsen et al. 2013; Sobo 2016). Indeed, parents think that the vaccinated are putting themselves or their children in danger. Second, while scientists and public officials are familiar with concepts like herd immunity, laypersons are largely unaware of them (Bradley and Navin 2021; Quadri-Sheriff et al. 2012; Sobo 2016). Vaccine refusers can hardly be subjective free-riders if they aren’t aware of the goods that benefit them. Third, vaccine refusers lack the motivational structure of free-riders. If vaccine refusers were free-riders, then we should be able to encourage vaccination by creating incentive structures that target the motivations of free-riders (Bradley and Navin 2021, p. 173). Since vaccine refusers aim to maximise their individual good by benefitting from a public good without paying, the thought goes that if we ensure that free-riding no longer maximises self-interest, then they would get vaccinated. They note, however, that coercive policies such as no jab, no pay; restricting access to public spaces; or even vaccine mandates, often increase, rather than eliminate, vaccine refusal (2021, p. 174).

Bradley and Navin’s first point that the reasons given for vaccine refusal often focus on safety and efficacy, not a desire to benefit from herd immunity, holds true for COVID-19 (Mascellino et al. 2021; Wouters 2021). However, on Cullity’s principle of unfairness, free-rider’s subjective attitudes are of little importance. Whether the vaccine provides a net benefit is an objective question. Therefore, false beliefs about the effectiveness of vaccines, or about covid, or the (unlikely) lack of awareness of relevant public goods, are irrelevant. We can make a comparison here to Vanderheiden’s discussion of climate change. A climate change denier may believe that it would harm their livelihood to waste money on green energy that is unnecessary. In this case, the climate change denier is just wrong about what is their net benefit because they are wrong about climate change. Likewise, a conspiracy theorist may believe that the government is using vaccinations to place tracking chips in people; thus, they have both a moral objection and an argument that vaccination is not a net benefit. However, given the falsity of the conspiracy theory, they misconceive their net benefit, so their objection is illegitimate.

Bradley and Navin might respond that this characterisation is unfair. “Recall that actual free-riders acknowledge the value of the public good they enjoy but refuse to pay the small cost of contributing to that good when they can get away with not incurring the cost” (2021, p. 174). It is unclear whether they mean that the free-rider must acknowledge their free-riding explicitly or privately. Given that the explicit definition would be obviously false, let’s take the latter interpretation. Even on this interpretation, one can be a free-rider and be unaware of it. Cullity provides a case in point (1995, p. 15). Suppose Sally goes to a new country that has a public policy like that in Fare Evasion, but Sally is unaware of this policy and so accidentally does not pay her fare. On Cullity’s understanding of unfair free-riding, Sally is still liable to pay for the fare, and liable for censure and punishment for not doing so. The mere fact that Sally was unaware of the policy is insufficient to render her innocent of free-riding; she benefits from a scheme that provides a net benefit to her without paying for it. We might morally excuse someone whose unfairness stems from ignorance rather than malice; however, it is unclear that one needs to be conscious of one’s unfairness to be unfair. Suppose that Sally is morally good and realises her mistake. She would recognise that she is already benefitting unfairly from the scheme, desire to pay the fare, and may apologise for her ignorance. She doesn’t become a free-rider only once she realises that she is benefitting from the scheme without paying her fair share and decides to keep doing that. Rather, she recognises that she is already free-riding and (if she is good), stops free-riding by paying the appropriate cost.

Bradley and Navin’s second point about laypersons lacking familiarity with herd immunity does not apply to COVID-19, at least not universally. For example, in the UK, herd immunity was initially touted in mainstream media and by the Prime Minister as a primary solution for getting out of the pandemic (BBC 2021). National rhetoric focused on the NHS as the provider of the public good of health, with slogans such as ‘protect the NHS’ making it clear that the NHS, something that most citizens regard as a valuable public good (Wellings 2017),^17^ is under threat and needs protecting. Finally, as everyone was subject to restrictions, they were trivially aware of those, too. Thus, at least with COVID-19, we can infer that the public had at least a basic sense of at least some of the relevant goods that are affected by COVID-19 and that can be protected or created by getting vaccinated.

Even if one disagrees with the empirical claim that laypersons are sufficiently aware, even in the case of COVID-19, of concepts like herd immunity, one can also disagree with Bradley and Navin on normative grounds. Typically, ignorance is not an excuse when it comes to moral failure, at least in cases where we can reasonably expect the agent to have known better (Rudy-Hiller 2022). I think such an argument can be applied (although not universally) to this case. Insofar as concepts like herd immunity and the need to protect public services were key public messages distributed by mainstream media platforms, it is reasonable to expect citizens to have at least some knowledge of the relevant concepts, and of the importance of contributing to goods such as herd immunity, the NHS, and freedom. This argument does not apply to everyone, for example, ethnic groups who do not speak English cite inability to access information in their native language as a primary reason for vaccine hesitancy (O’Shea et al. 2022, p. 21). Ignorance among such populations may be excusable, whereas for an average citizen whose media diet involves mainstream news, it is not excusable.

Let’s turn to Bradley and Navin’s third point about the motivational structure of free-riders. This argument presupposes simplistic and abstract notions of people’s motivations and incentives. They assume that if one appeals to the incentive structure of purported free-riders, and that they do not change their behaviour in the way that free-riders should, then they cannot be free-riders. However, one has not eliminated the possibility that vaccine refusal is caused by a rejection of coercive policies that the refuser deems unreasonable. Suppose June is an open free-rider. She openly admits that she benefits from herd immunity. The government appeals to her free-riding incentives by making it costly for her to free-ride. They implement a ‘no jab, no pay’ policy, and restrict her access to public spaces by implementing vaccine passports. Reacting to these policies, June remains unvaccinated in protest of policies that she believes undermine her rights. The fact that she doesn’t behave as a free-rider would is not because she wasn’t free-riding all along. Her motivations have changed because she believes that coercive vaccination policies undermine her human rights.

I agree with Bradley and Navin on one point concerning the subjective states of vaccine refusers. Insofar as refusers, because of their (false) beliefs or ignorance of the public benefits conferred by community vaccination, reject vaccination, it would be generally impracticable to condemn them as free-riders (2021, p. 176). This is because they will not see their behaviour, subjectively, as free-riding. Therefore, to treat them as such may exacerbate the problem. While this is an important prudential point, it does not show that vaccine refusers are not free-riders. Van den Hoven agrees that it may be unhelpful to call vaccine refusers free-riders, although she also argues that vaccine refusal is free-riding (2021, pp. 158–159). However, van den Hoven, Bradley and Navin focus on vaccine refusal generally, rather than on the specific emergency case presented by the COVID-19 pandemic.

I have responded to the subjective argument that vaccine refusers are not free-riders. First, vaccine refusers’ subjective motivations are largely irrelevant to whether they free-ride. Their false beliefs about COVID-19 vaccines cause them to misconceive what is a net benefit for them. Second, in the case of COVID-19, the public is typically aware of the many public goods to be gained through vaccination due to mainstream media messaging about concepts like herd immunity, protecting healthcare systems, and with respect to restrictions, being forced to follow them. Finally, we cannot draw easy conclusions about people’s subjective attitudes, given the complexity of human motivations and actions.

## Objection: vaccine refusal is free-riding, but not unfair

Marcel Verweij argues that although vaccine hesitancy is free-riding, it is not unfair. He construes fairness in terms of the distribution of the burdens and benefits of contributing to the public good in question. This position, which relies upon an argument for vaccine mandates on grounds of distributive fairness from Giubilini (2019, pp. 50–51), is that “the principle of fairness requires that any individual—at least those can who bear the (small) burdens [of vaccination]—should take their fair share in the fulfilment of the collective obligation. Of course, this only involves minor inconveniences and remote side effects, but still, it makes sense to distribute burdens and benefits in an equitable way” (Verweij 2022, p. 265).

Verweij claims that the principle of fairness is not violated in the herd immunity case. This is because contributors get the good of herd immunity as a free added extra to the cost of their individual immunity, which is got through vaccination. He writes: “If herd immunity constitutes a benefit at no extra cost or burden for contributors, then the distribution of burdens and benefits that results from the fact that some people take a free-ride, cannot be unfair (Verweij 2022, p. 237).

Verweij’s argument challenges condition (1) of Cullity’s principle of fairness. Although herd immunity is a public good, that good is a free extra benefit of individual vaccination. Therefore, the vaccinated cannot reasonably complain that the unvaccinated behave unfairly. Because the vaccinated are protected by being vaccinated, and herd immunity is a free extra, the distribution of burdens and benefits is not unfair.

I am unconvinced by Verweij’s argument. First, for many, the primary reason for receiving a COVID-19 vaccine is to create herd immunity, and it is the individual protection that is the free extra. Young people, children, and people without pre-existing conditions are less susceptible to serious COVID-19 symptoms and death. It is misleading to describe their contributions to herd immunity as mere extras when their primary aim is protecting their communities. Indeed, research on youth vaccination intentions shows that they are motivated by altruistic appeals to protect the most vulnerable, not self-interested appeals to protect their individual health (Rieger 2020; Tanaka et al. 2021). If we take their contributions seriously, then individual immunity is the free extra, while the primary benefit of these efforts is contributing to herd immunity.

Second, not everyone can benefit individually from vaccination. This is because the vaccines either are unsafe or ineffective for them. Such people have a legitimate complaint against free-riders who, as Verweij notes, threaten the protective seat that is meant for them (2022, p. 237). In this sense, one might construe free-riders as foul-dealers. Pettit describes foul-dealers as those who benefit from a scheme without paying for it at the expense of someone who contributes (Pettit 1986, pp. 371–372). Verweij can reply that vulnerable persons cannot complain because they don’t (because they can’t) contribute. They might have a complaint in terms of the *harm* that they may suffer, but this is a different complaint from the unfair free-riding complaint. We can respond by considering the indirect ways that even the unvaccintable can contribute to herd immunity, and the other public goods. To repeat, the good of herd immunity is a decrease in transmissibility and variants. Vaccination is the most direct way to achieve this benefit; however, it is possible to contribute to this good by observing social distancing and hygiene rules, and by regular COVID-19 testing. If an unvaccintable person contributes in this way, then they are doing their fair share in contributing to the public goods; therefore, they have a legitimate complaint against the unvaccinated on unfair free-riding grounds. One might respond that an unvaccinated person could also contribute in this way, and thus could also avoid the charge of free-riding. However, we should point out that they can still be criticised as they are taking are essentially paying a cost lower than their fair share. It would be like in *Fare Evasion*, if a person decided to only pay half of their fare despite being fully capable of paying the total. What makes acting short of vaccination reasonable for the vaccinatable is that they cannot contribute through vaccination.

Third, it isn’t clear that the notion of fairness that Verweij adopts is relevant to unfair free-riding. Even if herd immunity comes at no additional cost, the fact remains that herd immunity and the other public goods provide a net benefit to the vaccine refuser. These extra benefits would not exist without the efforts of the vaccinated. This seems to be the relevant moral issue, whether the benefit was gained as an added extra or not. Suppose that in *Private Road*, John’s neighbours tell the workmen not to tarmac John’s portion of the road so that he can’t benefit from their efforts; however, the kindly workmen fix John’s portion at no extra cost to the neighbours. John still free-rides, since it is only because his neighbours paid for the rest of the road that John could benefit in this way.

There is a fourth objection to Verweij which, though it arguably doesn’t apply to COVID-19, may apply in other cases of vaccine refusal.^18^ In some cases, refusal of some to vaccinate can contribute to a feedback loop, insofar as it may increase the need for further vaccines in the future. If an insufficient number of people get vaccinated to make use of herd immunity, then this increases the urgency for additional booster shots. Therefore, there is a legitimate complaint that non-free-riders can make to free-riders. It is the complaint that by refusing to vaccinate, these groups prolong the need for everyone else to get routine vaccinations. If everyone contributed, we may achieve herd immunity or even eliminate the infectious disease. Such an argument may not work for covid, due to the rapidity of mutations, though it works for cases where there is a reasonable expectation that, with sufficient vaccination, we can eliminate an infectious disease.

## Conclusion

In this paper, I have argued that COVID-19 vaccine refusal is unfair free riding. Their refusal is unfair because they should have contributed to the production of goods through vaccination as they provide a net benefit: protection of a social minimum that is threatened by the pandemic. Because the threat is of a basic social minimum and the costs of vaccination in terms of safety and time are insignificant, the goods are worth their cost. Moreover, the costs are distributed fairly as everyone is expected to make the same contribution in being vaccinated, with reasonable exceptions for the unvaccintable, and priorities for the vulnerable. A fair generalisation of a principle that requires cooperation with schemes that protect a social minimum or basic goods in emergencies does not leave practically everyone worse off. It would not require people to become vaccinated against every infectious disease; only those diseases which threaten a basic social minimum and that are emergencies. Finally, while there are moral objections against vaccination, many are either illegitimate or refutable.

## Footnotes

1 White (2021) is an example of a philosopher who does not make general conclusions about vaccine hesitancy as free-riding, tying them instead to a specific instance of hesitancy for a specific disease. This is the strategy I also adopt in this paper.

2 Free-riding can be viewed from a game theoretic perspective (Cullity 1995: 1). Such free-riding occurs when rational agents maximize their individual interests by not paying for a public good from which they benefit. Most discussion of vaccine hesitancy and free-riding uses the game theoretic characterisation (Bradley and Navin 2021, 2022; White 2021; Verweij 2022), though van der Van den Hoven (2012) provides an exception.

3 This follows Hart’s classics formulation: “When a number of persons conduct any joint enterprise according to rules and thus restrict their liberty, those who have submitted to these restrictions when required have a right to similar submission from those who have benefited by their submission” (1955: 185).

4 This is an adaptation of Cullity’s *Enterprising Elves* case from his paper. As his example is not an example of a collective good, I amended the example to suit my purposes. See Cullity (1995: 10) for his example.

5 He also discusses *congruent interests*, which refer to those things in which we have in the same kind of thing, though we can pursue and establish those interests individually; and *common interests*, which are those that we “necessarily and irreducibly share as a group or community” (Dawson 2011: 15–16).

6 Cullity’s remark here is for his original *enterprising elves* case, but his remark applies equally to my amended version.

7 Tampering could be morally acceptable: suppose the elves saw a toxic mould growing in my shoes that would kill me if they didn’t fix them. If you don’t think that any property has been tampered with, we can strengthen the intuition by imagining that the street is gated, with a sign saying “residents only” on it.

8 Cullity outlines six features of publicity (1995: 6–7).

9 Whenever we talk of a good’s being non-excludable, we must always specify the extent to which it is non-excludable, by pointing to the group or groups for whom it is non-excludable. For example, in private road, the good of a fixed road is a non-excludable good for all residents, since they all stand to benefit from a fixed road whether they pay for it or not, and it would be costly for the residents to try to exclude a resident from using it. However, the good is excludable with respect to non-residents, since it is easy for residents to exclude them from access.

10 If one thinks that herd immunity is in any way excludable, this only strengthens the argument here, which is that it is a non-excludable good. My own view is that exclusion is possible, but very costly.

11 A related point along these lines from Zameska (2021), is that participating in a vaccination scheme frees up health care resources that would otherwise be used to treat cases. Zameska calls the public good produced here ‘health systems capacity’ which is increased through vaccination (2021, p. 221).

12 Bradley and Navin respond that avoiding new variants is not necessarily a public good, since variants may be less dangerous than previous strains (2022: 168). They cite the Delta variant as an example. I reject this argument. We cannot know in advance whether a new variant will be more dangerous than the previous variant; therefore, it is still a greater benefit to slow or prevent new variants from spreading.

13 One might argue that the NHS is not non-excludable, as those without the right to reside in the UK are routinely excluded from it. However, recall that one must always specify the scope of non-excludability. For the NHS, that scope is arguably restricted to British citizens, who have the legal right to use the service and who have obligations to support the service through taxation and, in the case of Covid, getting vaccinated. Insofar as the NHS is non-excludable for British citizens, they are potential free-riders if they benefit from the good without supporting it; while those who are excluded would not be potential free-riders, as the good is excludable for them. Thank you to a reviewer for raising this worry.

14 White notes one such difficulty on moral grounds (2021, p. 4). It would be morally problematic to deny citizens access to health care if they need it just because they are unvaccinated. And it would be morally wrong to restrict their ability to access public spaces as well. However, as this point may stretch of the notion of “cost” too thinly, I include them here as tentative suggestions.

15 There are different interpretations of a social minimum: welfare interpretations focus on a minimum level of human happiness (Bentham 1789); the capabilities approach focuses on a defining a set of basic capacities which persons require to lead a minimally decent life (Sen 1992; Nussbaum 2000); or resources, which focuses on the means that people generally require to pursue their life plans (Rawls 1993: 187–190). For our purposes, we can remain open to any interpretation, since we can formulate the threats posed either in terms of a threat to happiness, capabilities, or resources.

16 See footnote 2 of this paper for a definition of prisoner’s dilemma free-riding.

17 This study found that 77% of participants belived that ‘the NHS is crucial to British society and we must do everything we can to maintain it’ (Wellings 2017).

18 Thank you to an anonymous reviewer for noting that this argument does not apply to our case.
